# Factors affecting the self-rated health of immigrant women married to native men and raising children in South Korea: a cross-sectional study

**DOI:** 10.1186/s12905-020-01073-8

**Published:** 2020-09-23

**Authors:** Bookyoung Kim, Kyung-Bok Son

**Affiliations:** 1 Seoul Sungwon Elementary School, Seoul, South Korea; 2 College of Education, Hanyang University, Seoul, South Korea; 3 College of Pharmacy, Ewha Womans University, 52 Ewhayeodae-gil, Seodaemun-gu, Seoul, 03760 Korea

**Keywords:** Immigrant woman, Foreign wife, Self-rated health, South Korea

## Abstract

**Background:**

Since the influx of international immigrants to South Korea (Korea) in the 1980s, the number of immigrants married to native Koreans has increased substantially over the last 30 years. This study aims to provide recent evidence on the self-rated health of immigrant women married to native men and raising children. We evaluated the self-rated health of immigrant women sorted by their country of origin and elucidated factors that affect their self-rated health.

**Methods:**

Data were obtained from the 2015 Korean National Multi-Cultural Family Survey. From the survey, a total of 6960 Korean-Chinese, Han-Chinese, Japanese, Vietnamese, and Filipino women were identified and a series of logistic regressions was conducted to elucidate factors that affected the self-rated health of immigrant women.

**Results:**

The majority of immigrant women in Korea perceived that they are healthy. However, the self-rated health of immigrant women varied by country of origin. Korean-Chinese and Japanese immigrants are less likely to perceive that they are healthy compared with Filipino and Vietnamese immigrants. We identified several factors at the individual, household, and community levels and found that the majority of them are likely to be ethnic dependent. However, satisfaction with husband and experience of unmet medical needs presented consistent results in the five ethnicity groups.

**Conclusions:**

Programs that strengthen spousal relationships and policies to enhance access to healthcare could be prioritized options to improve the self-rated health of immigrant women in Korea.

## Background

Since the influx of international immigrants to South Korea (Korea) in the 1980s, the number of immigrants married to native Koreans has increased substantially over the last 30 years. The total number of immigrants married to native Koreans was 155,457 in 2017, while it was only 93,786 in 2006 [1]. In terms of gender composition, the majority of immigrants married to native Koreans were female (approximately 85%). Marriage between Koreans and foreigners accounted for 10.6% of the 326,100 marriages registered in 2010; marriages between Korean men and foreign women and marriages between Korean women and foreign men accounted for 8.1 and 2.5%, respectively [1].

The family roles and responsibilities of female immigrants married to native men are essential [2–5]. Female immigrants play the roles of household keepers and mothers who stay at home and take care of their family members. Korean family life is based on the patriarchal system [6]: the husband is expected to be the head of the family and major decision maker, while the wife is expected to perform household tasks [6]. Thus, female immigrants married to native Korean men take responsibility for household tasks and spend more time on the primary care of children or ill family members than their husbands do. These traditional roles are challenged when female immigrants work outside the home to provide financial support for their families. However, they are still expected to perform household tasks.

Female immigrants married to native Korean men often decide to marry for financial reasons without understanding the culture of the new destination country or the communication expected with their new family members [7]. They experience difficulties related to the language, culture, values, and lifestyle of the new country or community, factors that are combined within the definition of acculturation in the literature [8, 9]. It has often been reported that acculturation is closely related to the health of immigrants [10, 11]. Furthermore, female immigrants experience marriage, pregnancy, childbirth, and nurturing in a relatively short period of time [10, 12]. Female immigrants typically lack knowledge of family planning and birth control [13], and they often give birth to a child during their first year of immigration [14, 15]. There are also many obstacles that prevent immigrant women from receiving sufficient antenatal care [16–18]. In other words, the immigrant women who have married native men and given birth to a child and are now raising children are in a vulnerable position from a health standpoint [19–22].

Given the increased number of foreign wives, their roles and responsibilities in a household, and the vulnerability of their health [19–22], immigrant women married to native men and raising children in Korea have been the subject of considerable interest from the perspective of society. In a similar vein, several policies and programs, entitled “Multicultural Policies”, have been introduced in Korea to aid their adaptation and integration into society [23]. The Korean government have legislated the “Act on the Treatment of Foreigners in Korea” and “Support for Multicultural Families Act”, implemented social integration policies, and assigned (relatively large) budgets for policies and programs to support multicultural families.

Understanding the health status of immigrant women and factors affecting the self-rated health of immigrants may offer guidance for the government to assist them in adapting healthily and successfully to their new environment. However, little is known about the health of immigrant women, particularly for those who married native men, gave birth to a child, and are now raising children in Korea [19, 21]. This study aims to provide recent evidence on the self-rated health of immigrant women. We evaluated the self-rated health of immigrant women sorted by their country of origin and elucidated factors that affect their self-rated health. Taken together, we suggest policy options to improve the health of immigrant women in Korea. In particular, this study was designed to address the following research questions:

1. What is the level of self-rated health of immigrant women married to native men and raising children?
2. What are the associations between self-rated health and the variables at the individual level, including current age, age at arrival, educational attainment, subjective social status, residency area, ethnicity, and fluency in Korea?
3. What are the associations between self-rated health and the variables at the household level, including immigrant women’s satisfaction with their husbands and children?
4. What are the associations between self-rated health and the variables at the community level, including the experience of discrimination and unmet medical needs?
5. What is the relationship between the country of origin and factors affecting self-rated health?

## Methods

### Subjects

**Fig. 1:**
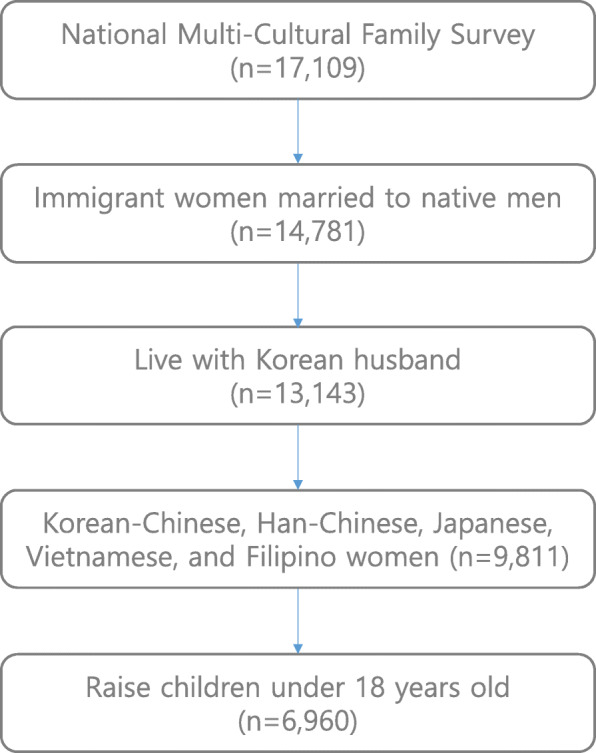
Sampling process for this study

The subjects of this study were immigrant women who were married to native men and had children under 18 years old in Korea. This study limited the inclusion of subjects by their country of origin, and included Korean-Chinese, Han-Chinese, Japanese, Vietnamese, and Filipino women for the analysis. In the end, 6960 eligible subjects were obtained among 17,109 married immigrants or naturalized persons (Fig. 1).

### Data source

This was a cross-sectional study. We used the data from the Korean National Multi-Cultural Family Survey (NMFS). The survey was first conducted by the Ministry of Health and Welfare to investigate the living conditions and welfare needs of multicultural families in 2009 [24]. The sample of the NMFS consists of 27,120 households with married immigrants or naturalized persons, representing approximately 10% of total multicultural households in Korea [24]. Information on multicultural families for the NMFS was provided by the Ministry of Public Administration and Security, and then trained interviewers visited households and conducted the survey, which was offered in 10 different languages. The questionnaire collected information on personal background, employment, marriage and family relations, and social life. In the 2015 NMFS survey, 17,109 married immigrants or naturalized persons (63%) responded to the survey, indicating that the survey is likely to be representative [24]. Various empirical studies based on the NMFS survey have been published in many research areas, including social services [25] and health care [26].

### Variables and measurement

#### Dependent variable

To capture the health status of subjects, we used self-rated health as the dependent variable. Self-rated health is a simple and valid method to measure health status [27–29], and allows respondents to prioritize and evaluate various aspects of their health from physical to mental health [30]. It is a reliable method to measure health among those without cognitive impairment [31–34]. Self-rated health has been at least moderately associated with physician assessments of health [35]. Thus, it frequently replaces clinical assessments in survey literature. The survey asked the subjects “What is your general health condition?”, which they rate on a five-point Likert scale (very well = 1, well = 2, neutral = 3, bad = 4, and very bad = 5). For this study, the variable was used as binary, either good (very well, well) or poor (neutral, bad, very bad).

#### Independent variables

We used various independent variables at the individual, household, and community levels. At the individual level, we used sociodemographic variables, including current age (continuous), age at arrival (continuous), country of origin, educational attainment, subjective social status (continuous), residency area, and fluency in Korean (continuous). Age at arrival was calculated by subtracting the date of birth from the date of immigration. Educational attainment was categorized into less than 9 years, 9–12 years, and more than 12 years. Subjective social status was used as a continuous variable (low = 1, middle = 2, high = 3). In the NMFS, fluency in Korean was measured on a five-point Likert scale (very good = 1, good = 2, neutral = 3, bad = 4, very bad = 5) from the perspectives of speaking, listening, reading, and writing. Based on this information, we calculated the average score and categorized the fluency of Korean into three levels: poor, average, and good.

We added variables at the household and community level in the model. As previously mentioned, this study was interested in the health status of immigrant women who married native men and had children under 18 years old in Korea. Thus, we used variables regarding satisfaction with husband and children (continuous with very bad = 1 and very good = 5) and the number of children under 5 years old (continuous) in the model. Finally, we added experience of discrimination in general and unmet medical needs of immigrant women as binary variables in the model at the community level.

### Statistical analysis

Descriptive analyses were used to examine the self-rated health of immigrant women. Specifically, *chi-square* tests for the categorical covariates and *t* tests for the continuous covariates were conducted to examine whether variables of interest differed by group, including good and poor self-rated health. A series of logistic regressions was conducted to elucidate factors that affected the self-rated health of immigrant women. Model 1 is the simplest model which was adjusted for variables at the individual level. Model 2 was adjusted for the variables at the individual and household levels, and Model 3 is the fully adjusted model, which includes all the variables at the individual, household, and community levels. Furthermore, we separated the subjects by country of origin and applied the fully adjusted model to capture different factors that affect the self-rated health of immigrant women within the ethnicity group. Data management and analyses were performed using R statistical software (version 3.4.1). Statistical significance was defined as *p*-values less than 0.05.

## Results

### Characteristics of the subjects

Table 1 shows the characteristics of the subjects divided into two groups, namely, those with good and poor self-rated health. Among the 6960 subjects, 4663 subjects (67%) reported that they were in good health. Significant differences were noted in the variables of current age, age at arrival, education, subjective social status, ethnicity, fluency in Korean, satisfaction with husband and children, number of children under 5 years old, and the experience of discrimination and unmet medical needs between the two groups. The mean age of the subjects was 33.3 years (standard deviation 7.29), and the mean age at arrival of the subjects was 24.6 years (5.11) in the group who responded that they were healthy. Those variables were 36.9 years (8.65) and 26.1 years (5.72), respectively, for the subjects in the group who responded that they were not healthy. Differences in satisfaction with husband and children were also noted between the two groups. The score for satisfaction with husband was higher for the healthy group than for the unhealthy group (4.03 versus 3.54). The score for satisfaction with children was also higher for the healthy group than for the unhealthy group (4.54 versus 4.26). It is notable that the score for satisfaction with children was higher than the score for satisfaction with husband in both groups. Finally, the subjects in the healthy group were less likely to experience discrimination and unmet medical needs than the subjects in the unhealthy group: 35% versus 48 and 7% versus 19%, respectively.

**Table 1:** Descriptive characteristics of the subjects

	Good (*n* = 4663)		Poor (*n* = 2297)		*p*-value	
Age (mean and SD)	33.3 (7.29)		36.9 (8.65)		17.15	*P* < 0.001
Age at arrival	24.6 (5.11)		26.1 (5.72)		10.78	*P* < 0.001
Education						
Under 9 years	1196	26%	580	25%	14.80	*P* < 0.001
9–12 years	2011	43%	1091	47%		
Above 13 years	1456	31%	626	27%		
Subjective social status						
Low	1094	23%	861	37%	179.29	*P* < 0.001
Middle	3320	71%	1395	61%		
High	249	5%	41	2%		
Residency area						
Urban	2762	59%	1346	59%	0.23	0.6313
Rural	1901	41%	951	41%		
Ethnicity						
Han-Chinese	1255	27%	570	25%	157.81	*P* < 0.001
Vietnamese	1543	33%	673	29%		
Korean-Chinese	449	10%	294	13%		
Filipino	850	18%	264	11%		
Japanese	566	12%	496	22%		
Fluency in Korean						
Poor	354	8%	181	8%	57.91	*P* < 0.001
Average	1948	42%	1170	51%		
Good	2361	51%	946	41%		
Satisfaction with husband	4.03 (0.92)		3.54 (1.01)		−19.39	*P* < 0.001
Satisfaction with children	4.54 (0.69)		4.26 (0.85)		−13.86	*P* < 0.001
Number of children under 5 years						
0	1617	35%	1156	50%	161.7	*P* < 0.001
1	2188	47%	858	37%		
2	796	17%	265	12%		
3≤	62	1%	18	1%		
Experience of discrimination						
No	3030	65%	1200	52%	104.2	*P* < 0.001
Yes	1633	35%	1097	48%		
Experience of unmet medical needs						
No	4356	93%	1856	81%	254.0	*P* < 0.001
Yes	307	7%	441	19%		

Table 2 presents the characteristics of subjects sorted by ethnic group. The self-rated health of immigrant women varied by country of origin. The proportion of subjects who responded that they were healthy was, in order, Filipino (76%), Vietnamese (70%), Han-Chinese (69%), Korean-Chinese (60%), and Japanese (53%). Filipino immigrant women were more likely to perceive themselves as healthy, while Korean-Chinese and Japanese immigrant women were less likely to respond that they were healthy. The average age at the time of the survey and the average age at arrival also varied by country of origin. The average age at the time of the survey of Vietnamese women was 28.49 years old, while that of Japanese women was 43.42 years old. Similarly, the average age at arrival of Vietnamese women was 22.05 years old, while that of Japanese women was 29.23 years old. Given these results on the age and income level of the country of origin, we could assume that Japanese women had higher educational attainment and were less likely to be raising children under 5 years old, while Vietnamese women had lower educational attainment and were more likely to be raising children under 5 years old.

**Table 2:** Descriptive characteristics of the subjects sorted by country of origins

	Han-Chinese *N* = 1825		Vietnamese *N* = 2216		Korean-Chinese *N* = 743		Filipino *N* = 1114		Japanese *N* = 1062	
Self-rated health										
Poor	570	31%	673	30%	294	40%	264	24%	496	47%
Good	1255	69%	1543	70%	449	60%	850	76%	566	53%
Age	35.64 (6.02)		28.49 (4.86)		37.92 (5.98)		33.75 (7.61)		43.42 (7.11)	
Age at arrival	25.86 (5.29)		22.05 (4.26)		26.07 (5.39)		25.10 (4.71)		29.23 (4.59)	
Education										
Under 9 years	398	22%	1117	50%	157	21%	92	8%	12	1%
9–12 years	924	51%	946	43%	426	57%	426	38%	380	36%
Above 13 years	503	28%	153	7%	160	22%	596	54%	670	63%
Subjective social status										
Low	547	30%	591	27%	251	34%	316	28%	250	24%
Middle	1207	66%	1525	69%	471	63%	753	68%	759	71%
High	71	4%	100	5%	21	3%	45	4%	53	5%
Residency area										
Urban	1115	61%	1191	54%	518	70%	608	55%	636	60%
Rural	670	37%	1025	46%	225	30%	506	45%	426	40%
Fluency in Korean										
Poor	136	7%	213	10%	9	1%	103	9%	74	7%
Average	533	29%	1386	63%	85	11%	655	59%	459	43%
Good	1156	63%	617	28%	649	87%	356	32%	529	50%
Satisfaction with husband	3.90 (1.01)		3.83 (0.94)		3.93 (0.98)		3.96 (0.96)		3.77 (1.00)	
Satisfaction with children	4.46 (0.74)		4.55 (0.69)		4.40 (0.78)		4.55 (0.69)		4.12 (0.87)	
Number of children under 5 years										
0	790	43%	499	23%	414	56%	415	37%	655	62%
1	788	43%	1231	56%	272	37%	496	45%	259	24%
2	236	13%	457	21%	52	7%	185	17%	131	12%
3≤	11	1%	29	1%	5	1%	18	2%	17	2%
Experience of discrimination										
No	1099	60%	1321	60%	456	61%	681	61%	673	63%
Yes	726	40%	895	40%	287	39%	433	39%	389	37%
Experience of unmet medical needs										
No	1636	90%	2003	90%	673	91%	990	89%	910	86%
Yes	189	10%	213	10%	70	9%	124	11%	152	14%

At the household level, the score for satisfaction with husband and children varied by country of origin. The satisfaction with husband and children scores were lower for Japanese women than for the remaining groups. The satisfaction scores of Japanese women were 3.77 and 4.12 for their husband and children, respectively, while the scores of the variables were 3.96 and 4.55, respectively, for Filipino women. At the community level, the experience of discrimination and unmet medical needs also varied by country of origin. Japanese women were less likely to report discrimination and more likely to report unmet medical needs when compared with the results of the remaining group. For instance, 37 and 14% of Japanese women reported discrimination and unmet medical needs, respectively, while 39 and 11% of Filipino women reported discrimination and unmet medical needs, respectively.

### Factors affecting the self-rated health of female immigrants

Table 3 presents the logistic regression for the different models. Model 1, adjusted for the variables at the individual level, shows that age at arrival, subjective social status, and fluency in Korean were positively associated with their self-rated health, while current age was negatively associated with their self-rated health. The association between these variables and self-rated health was consistent in Models 2 and 3. In Models 2 and 3, we found that satisfaction with husband and children was positively associated with self-rated health at the household level, and the experience of discrimination and unmet medical needs were negatively related to health status at the community level.

**Table 3:** Regression models of self-rated health of immigrants women

	Model 1 (*N* = 6960)			Model 2 (*N* = 6960)			Model 3 (*N* = 6960)		
	Estimate	Std.error	*p* value	Estimate	Std.error	*p* value	Estimate	Std.error	*p* value
Individual									
Age	−0.084	0.006	***	−0.066	0.007	***	−0.071	0.008	***
Age at arrival	0.040	0.007	***	0.028	0.008	***	0.031	0.008	***
Education (Ref_under 9 years)									
9–12 years	−0.070	0.069		−0.106	0.071		−0.118	0.072	
Above 13 years	0.159	0.087		0.072	0.088		0.085	0.091	
Subjective social status	0.573	0.053	***	0.407	0.055	***	0.349	0.056	***
Residency area	0.014	0.055		−0.008	0.056		− 0.056	0.057	
Ethnicity (Ref_Han-Chinese)									
Vietnamese	−0.229	0.082	**	−0.191	0.084	*	−0.226	0.085	**
Korean-Chinese	−0.316	0.095	***	−0.349	0.097	***	−0.361	0.099	***
Filipino	0.407	0.094	***	0.398	0.096	***	0.396	0.097	***
Japanese	−0.225	0.096	*	− 0.188	0.099		−0.172	0.101	
Fluency in Korean	0.493	0.048	***	0.428	0.049	***	0.395	0.050	***
Household									
Satisfaction with husband				0.362	0.029	***	0.319	0.030	***
Satisfaction with children				0.222	0.037	***	0.216	0.037	***
Number of children under 5 years				0.047	0.046		0.049	0.047	
Community									
Experience of discrimination							−0.396	0.057	***
Experience of unmet medical needs							−1.028	0.087	***

* *p* < .05; ** *p* < .01; *** *p* < .001

Table 4 presents the results of the logistic regression sorted by ethnic group. In Table 3, we found the association between self-rated health and the variables on age, age at arrival, subjective social status, and fluency in Korean at the individual level, the variables on satisfaction with husband and children at the household level, and the variables on experience of discrimination and unmet medical needs at the community level. However, the association between self-rated health and the variables was not consistent when we applied the separated model sorted by ethnic group. Satisfaction with children at the household level was not significantly associated with the health status of Vietnamese women. Experience of discrimination at the community level was not significantly associated with the health status of Japanese women. Subjective social status at the individual level was not significantly associated with the health status of Vietnamese and Japanese women. However, it is noteworthy that satisfaction with husband and the experience of unmet medical needs were consistent factors in all ethnic groups.

**Table 4:** Regression models of self-rated health of immigrants women sorted by country of origins

	Han-Chinese *N* = 1825			Vietnamese *N* = 2216			Korean-Chinese *N* = 743			Filipino *N* = 1114			Japanese *N* = 1062		
	Estimate	Std.error	*p* value	Estimate	Std.error	*p* value	Estimate	Std.error	*p* value	Estimate	Std.error	*p* value	Estimate	Std.error	*p* value
Individual															
Age	−0.107	0.015	***	−0.017	0.018		−0.042	0.025		−0.107	0.015	***	−0.053	0.017	**
Age at arrival	0.058	0.015	***	−0.002	0.019		−0.023	0.025		0.058	0.015	***	0.018	0.019	
Education (Ref_under 9 years)															
9–12 years	0.006	0.139		−0.118	0.102		0.013	0.214		0.006	0.139		−0.821	0.678	
Above 13 years	0.391	0.167	*	−0.021	0.202		−0.002	0.266		0.391	0.167	*	−0.679	0.678	
Subjective social status	0.545	0.111	***	0.135	0.097		0.599	0.169	***	0.545	0.111	***	0.285	0.153	
Residency area	−0.184	0.115		−0.011	0.098		0.187	0.185		−0.184	0.115		− 0.085	0.149	
Fluency in Korean	0.317	0.096	***	0.313	0.087	***	0.365	0.225		0.317	0.096	***	0.785	0.127	***
Household															
Satisfaction with husband	0.249	0.057	***	0.463	0.055	***	0.201	0.090	*	0.249	0.057	***	0.423	0.079	***
Satisfaction with children	0.244	0.074	**	−0.036	0.070		0.340	0.108	**	0.244	0.074	**	0.458	0.089	***
Number of children under 5 years	−0.088	0.095		−0.024	0.077		0.213	0.159		−0.088	0.095		0.261	0.133	
Community															
Experience of discrimination	−0.712	0.114	***	−0.324	0.099	**	−0.504	0.173	**	−0.712	0.114	***	−0.213	0.149	
Experience of unmet medical needs	−0.942	0.174	***	−0.982	0.153	***	−1.929	0.341	***	−0.942	0.174	***	−0.771	0.217	***

* *p* < .05; ** *p* < .01; *** *p* < .001

## Discussion

Despite the growing number of immigrant women and their dual burden as foreign wives, including acculturation and the rapid change in family roles and responsibilities, there has been a lack of studies examining the health status of immigrant women in Korea. This study evaluated the self-rated health of immigrant women who experienced acculturation, marriage, pregnancy, childbirth, and nurturing in a relatively short period of time [10, 36], and analyzed factors that affected the health status of immigrant women using a national survey. In this chapter, we discussed the self-rated health of immigrant women, the association between self-rated health and the selected variables, and policy options to improve the health of immigrant women.

### Self-rated health of immigrant women

In the 2015 NMFS survey, we found that 67% of subjects who were married to native men and had children under 18 years old responded that they were in good health. It is interesting to compare the value reported in this study with other literature. Lee and Kim (2014) reported the self-rated health of foreign women aged 18 to 59 who were married to native men using the 2009 NMFS survey [37]. Among the 57,838 subjects, 51,995 subjects (90%) responded that they were in good health. The proportion of subjects who responded that they were healthy was, in order, Vietnamese (94%), Filipino (93%), Han-Chinese (89%), Japanese (88%), and Korean-Chinese (86%).

The variance in the results, 67% versus 90%, could be explained by two factors: the study population and definition of good health. First, this study has interest in the health of female immigrants who married native men and were raising a child or children. Thus, we exclude female immigrants who did not give birth to a child or who do not care for a child or children. Second, this study defined good health as when the subjects responded that their general health condition was very good or good. However, Lee and Kim (2014) defined good health when the subjects responded with very good, good, or neutral [37]. Even though there were differences in the study population and the definition of good health, we could observe that Filipino and Vietnamese immigrants are more likely to perceive that they are healthy, while Korean-Chinese and Japanese immigrants are less likely to perceive that they are healthy.

### Associations between self-rated health and the variables of interest

In this study, we identified various factors that might affect the self-rated health of immigrants at the individual, household, and community levels and applied logistic models to examine the association between self-rated health and the variables of interest.

The self-rated health of immigrants worsens as they age [24]. We found that age was negatively associated with the health status of the subjects, implying that the health status of aged immigrants women should be closely monitored [38]. This study also demonstrated that increased age at arrival was associated with better self-rated health, which is not a common observation in other countries [11, 39]. African American immigrants in the United States showed a negative association between increased age at arrival and self-rated health [11]. Foreign wives [40] who came to Korea at a younger age tended to be from poorer households or less-developed countries compared with those who came to Korea at an older age. For instance, the average age of arrival of Vietnamese women was 22.05 years, and their educational attainment was low, while that of Japanese women was 29.23 years, and their educational attainment was high.

Acculturation is often conceptualized as a process by which an immigrant adopts the language, culture, values, and lifestyle of the new country [20]. Previous studies have found that language fluency is associated with the health status of immigrants. African American immigrants with greater fluency in English were more likely to be healthier [11]. Female immigrants with greater language fluency tend to adapt more successfully to their marriage and to have better mental health in other studies conducted in Korea [41, 42]. Similar to those studies, this study supports that language proficiency is associated with better self-rated health. However, fluency in Korean was not a significant variable in explaining the self-rated health of Korean-Chinese immigrant women. Korean-Chinese women are more fluent in Korean than other ethnicity groups, implying that Korean-Chinese women may suffer less from differences in languages compared with other groups.

The eligible subjects in this study experienced marriage, pregnancy, childbirth, and nurturing in a relatively short period of time [10, 13]. They might have felt lonely during pregnancy because they could not talk with friends and family from their country of origin. Furthermore, immigrant women typically began to learn Korean during pregnancy, which occurred within 1 year of immigration or marriage, and were not fluent in Korean, making it more difficult to be socialized in the community. Immigrant women relied on their husbands during this period, and they embraced their role as mothers when the baby arrived [15]. We adopted these speculations in our empirical model and found that immigrant women’s relationships with their husbands and children were closely related to their health status. These findings imply that programs that aim to promote the health of female immigrants should also pay attention to their relationships with their husbands and children.

Discrimination has been conceptualized as judgment or treatment on the basis of prejudice against racial and ethnic membership [43, 44]. The literature from various backgrounds and contexts have presented that there is a significant relationship among perceived discrimination, psychological distress, adjustment, and health outcomes [45–50]. This study confirmed that perceived discrimination had a negative association with the self-rated health of immigrant women. However, it is noteworthy that perceived discrimination and its association with self-rated health is likely to be an ethnic-dependent variable. Japanese women were less likely to perceive discrimination, and their perceived discrimination was not significantly related to self-rated health.

### Policy options to improve the health status of immigrant women

We found interesting results on the self-rated health of immigrant women. First, we found that the self-rated health of immigrant women varied by country of origin. In particular, Korean-Chinese and Japanese immigrants are less likely to perceive that they are healthy compared with Filipino and Vietnamese immigrants. Second, we identified several factors at the individual, household, and community levels and found that the majority of them are likely to be ethnic dependent. However, it is noteworthy that the factors satisfaction with husband and experience of unmet medical needs presented consistent results in the five ethnicity groups. Based on these observations, we suggest general policy options to improve the health of immigrant women.

In our analysis, we confirmed that unmet medical needs were significantly related to the self-rated health of immigrant women. Thus, policies to enhance access to healthcare could be prioritized to improve the health of immigrant women in Korea. Access to healthcare is one of the biggest challenges that migrants face in their host communities. Migrants often face formal or informal barriers in accessing health services [16–18]. Language barriers, lack of knowledge about diseases and availability of services, insufficient health literacy, and financial hardships might be barriers to access to healthcare. Thus, it has been reported that disparities in healthcare utilization exist between immigrants and nonimmigrants [51]. However, recently published literature presented that disparities in healthcare utilization vary according to the types of healthcare services [17]. Utilization of emergency services and hospitalization were higher among migrants compared with nonimmigrants, while outpatient visits for specialized care were lower among migrants compared with nonimmigrants. Literature on the disparity in healthcare utilization between immigrants and nonimmigrants is scarce in Korea. However, immigrant women received fewer healthcare services during pregnancy than Korean native women [36], even though the Korean government has enacted laws that require the government to educate immigrant women and provide perinatal care and medical checkups during pregnancy [52].

Policies to strengthen spousal relationships could be the other prioritized option to improve the self-rated health of immigrant women. Satisfaction with husband also presented a consistent association with the self-rated health of immigrant women. Immigrant women faced dual burdens as foreign wives: acculturation and rapid change in family roles. Furthermore, they are exposed to multiple social risks, such as social isolation and violence [53]. Marriage satisfaction and social support are protective factors for immigrant women to address the burden that foreign wives experience. In particular, successful adaptation to marriage and a satisfactory marriage with a Korean husband are linked to the self-rated health of immigrant women [22].

### Study limitations

This study had several limitations. First, we applied a cross-sectional study, which may hinder the interpretation of the results as a causal relationship. Second, this study used the self-rated health of immigrants to measure health status. Sometimes, self-rated health allows respondents to measure different aspects of their health, from physical to mental health. Therefore, asking different questions regarding health status, such as whether a chronic disease is present, is needed to complement self-rated health. In a similar vein, different cultural manifestations and expressions of health or healthcare have been documented in the literature [54–57], implying that intercountry comparisons of self-rated health are very difficult to interpret. Furthermore, language or translation in conducting surveys based on intercountry comparisons might cause a potential limitation [58, 59]. Last, this study noted the countries of origin of immigrant women and conducted a subgroup analysis to understand variations in ethnicity groups. Further longitudinal design and qualitative methods are needed to understand the mechanisms of cultural diversity and countries of origin on the health status of immigrant women.

## Conclusion

The majority of immigrant women in Korea perceived that they are healthy. However, the self-rated health of immigrant women varied by their country of origin. In particular, Korean-Chinese and Japanese immigrants are less likely to perceive that they are healthy compared with Filipino and Vietnamese immigrants. We presented various factors that might affect the self-rated health of immigrants at the individual, household, and community levels. At the individual level, we found that age at arrival, subjective social status, and fluency in Korean were positively associated with their self-rated health, while current age was negatively associated with their self-rated health. We also found that satisfaction with husband and children was positively associated with self-rated health at the household level, and the experience of discrimination and unmet medical needs were negatively associated with health status at the community level. Most of the variables were ethnic-specific factors that affect self-rated health of a certain ethnicity. However, satisfaction with husband and experience of unmet medical needs presented consistent results in the five ethnicity groups. These observations imply that programs that strengthen spousal relationships and policies to enhance access to healthcare could be prioritized options to improve the self-rated health of immigrant women in Korea.

## Data Availability

The datasets used during the current study are available from the corresponding author on reasonable request.

## Abbreviations

Korea: South Korea
NMFS: National Multi-Cultural Family Survey
